# Ethical Issues in Care and Treatment of Neuronal Ceroid Lipofuscinoses (NCL)–A Personal View

**DOI:** 10.3389/fneur.2021.692527

**Published:** 2021-06-25

**Authors:** Alfried Kohlschütter

**Affiliations:** Department of Pediatrics, University Medical Center Eppendorf, Hamburg, Germany

**Keywords:** dementia, genetic, lysosomal storage disease, children, palliative medicine, disease-modifying therapy, newborn screening

## Abstract

The management of Neuronal Ceroid Lipofuscinoses (NCL), a group of genetic neurodegenerative disorders mainly affecting brain and retinas, raises difficult questions for physicians and other professionals in research, pharmaceutical industry, and public health. Ethical problems in medicine cannot be solved by rational deliberation or by following formal rules. Two topics of ethical issues in the field of NCL are presented here. One group relates to the care of individual patients and centers on a life with dementia at a young age. Advanced care planning for the end of life and the use of life-prolonging measures require challenging assumptions in the best interest of a patient. A second group of questions relates to new treatments. Impressive novel putative causal therapies, such as enzyme replacement for CLN2 disease, may be only disease-modifying and carry the risk of changing a deadly disease of short duration into one with prolonged survival and poor quality of life. The wish for better therapeutic interventions in life-limiting diseases has to take such risks, but more experience is needed before definite conclusions can be drawn. The appropriateness of presymptomatic screening for a severe disease, e.g., must be carefully evaluated to avoid the disastrous experience made with the rash start of newborn screening for Krabbe disease. The ethical issues described and commented in the article reflect the personal experience of a pediatrician who has studied clinical and research questions in NCL for four decades. They should alert various professionals to the necessity of taking their own decisions in situations that are caused by rare progressive brain diseases of young persons, as typified by the NCL.

## Introduction

Ethical problems in medicine are questions that cannot be answered rationally or by following standardized rules. Answers have to evolve on the basis of a personal attempt to come to terms with an ethical dilemma. Neuronal Ceroid Lipofuscinoses (NCL), a group of genetic life-limiting neurodegenerative disorders predominantly affecting the brain and retina of children, raise questions that concern physicians involved in patient care as well as other professionals in research, pharmaceutical industry, and public health.

This review reflects the personal experience of a pediatrician who has been dealing with NCL families and research for four decades. Ethical issues encountered are presented in two sections: questions related to the care of patients and more general questions with impact on other areas of healthcare. The article should allow readers to recognize responsibilities of various professionals taking care of NCL and show ways along which decisions can be reached.

## Aspects of a Life With Dementia in the Young

The different NCL, named after their defects of the genes *CLN1* to *CLN14* (1), display a high clinical variability and can manifest from birth to young adulthood (2). All forms of NCL lead to dementia, helplessness and premature death. To understand the personality and the psychosocial situation of a NCL patient, one has to realize that the individual had been healthy and developed normally before the onset of symptoms (with the exception of congenital cases), that the clinical deterioration is inexorably progressive, and that the patient and the family are repeatedly confronted with terrifying and demanding situations.

The authorization of a physician to decide on ambiguous ethical questions is derived from a thorough understanding of a patient's condition and from respecting the individuality of the situation. Apart from these prerequisites and consideration of the general principles of medical ethics [beneficence, non-maleficence, autonomy, and justice (3)], little else is needed for ethical deliberations of a responsibly acting physician.

### Balancing Patient Autonomy and Dementia

A demented person unable to communicate verbally may still be able to express a judgement on his or her own situation. The story of a young man told in Box 1 illustrates this poignantly.

Box 1 **A resolute personality.**

Arthur (not his real name) was diagnosed with juvenile CLN3 disease as a first grader after his vision had deteriorated. He was transferred to a school for the visually handicapped. Gradually, he lost intellectual capacity and developed seizures but remained a strong boy, good-humored when he got his ways and fighting fiercely against everything he did not like. His parents acquainted themselves with the dire prognosis and concluded that, should their son become helpless, any measure to prolong life unnaturally would appear to them entirely inappropriate. They asked if I, as the familiar specialized pediatrician, would stand by them should such a situation arise. I kept seeing Arthur from time to time.

Arthur ended up helpless, unable to converse verbally and being cared for in an excellent nursing home. When he was 27, his parents, still in custody, called me because he had started, without comprehensible reason, to refuse feeding and reacted fiercely to any attempt of offering food. Multidisciplinary examination did not reveal an organic explanation. When I saw him, he was in good spirits and reacted in a friendly way. When offered a spoonful of a favorite food, he vehemently turned away his head. His parents assumed his behavior reflected that Arthur was “fed up” with life. I examined him, observed him for a long time, and collected additional information from persons who knew him. One of his teachers commented that he had always reacted in such a way when something went against his will.

In the end, I concluded that the parents' interpretation was probably right and that the arguments for starting artificial nutrition were less weighty than those for withholding it. Consensus of opinion with the nursing team was reached and feeding was discontinued. Arthur's mouth was kept moist. He remained quiet without signs of discomfort. A few days later, he developed fever and a cough, and subsequently pneumonia. When he appeared to have difficulty breathing, he was given oxygen and eventually morphine. Shortly afterwards, he died. Arthur's death certificate read “natural death.”

*Comment. The ethical problem was deciding whether an indication existed for starting artificial nutrition, an invasive intervention that must have a treatment goal, must be supported by scientific evidence, and requires patient consent* *(**4**). In this case, indisputable evidence was absent, the patient*'*s will could only be guessed from non-verbal signals, and a decision had to be made together with parents on the basis of the personal judgment of a physician familiar with the patient*'*s situation (“shared decision making”)* *(**5**). In some countries, it is not legal to withdraw artificial nutrition in terminally ill patients as it is not considered a medical intervention but a component of basic care* *(**4**). In these countries, it might be even more difficult to reach a balanced decision*.

“*Consensus with the nursing team”: For problems as described in* *Box 1**, multidisciplinary discussion and shared decision making is usually advised. Consensus of the multiprofessional team is of great importance for sustained patient care. While various involved healthcare professionals may have different helpful arguments and should be heard, the final responsibility should rest on a single physician*.

### Vital Decisions in the Patient's Best Interest

Choices of adults regarding the end of their lives are liberally discussed at present, while legal frameworks remain controversial (6, 7). Decision-making capacities of children and decisionally vulnerable adults are even more delicate, but must not be neglected (8, 9).

There is wide consensus that vital medical decisions should be taken in the best interest of the patient. Defining this best interest is difficult against the background of cognitive impairment and in children. Substitutes must therefore form a proper idea of a patient's best interest. In children, the natural substitutes are their parents. For them, this assignment can be a severe burden that requires great emotional support and understanding of the consequences of a decision. Supporting the parents, informing and “educating” them appropriately (which typically requires many repeated discussions) is a demanding job for the doctor in charge.

Sometimes, accepting death as a consequence of a decision may be in the best interest of the patient. A pediatrician will have to make up his own mind about the patients' wish and then try to make the parents understand what is at stake. When the physician succeeds with this, outside views coming from the family and from their environment may further complicate the situation. For a discussion of the best-interests-of-the-child framework, see Engelhardt (10) and Zawati et al. (11).

Ideally, even parents as medical lay persons can appropriately decide in difficult medical matters (see Box 1), provided they are adequately informed and understand the consequences, not only in respect to their child's life but also in regard to their further existence in their social world.

Advice can be given using differing attitudes, more paternalistic or more objective, depending on the intellectual abilities and psychological circumstances. When consensus exists between both parents and their physician, a vital decision can be made without sharing responsibility with persons outside the family. If this is not possible in exceptional cases, advice must be sought from other sides, which may include institutional ethical review boards. External advisers, however, should not give the impression that they are taking a final decision. They should rather facilitate discussion of arguments and help parents find a decision they can live with. For further discussion, see Hain (12).

*Comment: Measures or interventions to be taken or not in critical situations to be expected must be discussed well in advance. At an international workshop with participation of patient families, it was concluded that decisions on life-prolonging interventions in children with advanced NCL or other degenerative brain diseases are highly individual but can be made in a rationally and emotionally acceptable way* *(**13**)*.

### Quality of Life

In the case of a helpless, non-communicative person with an incurable life-limiting disease, speculations about the patient's quality of life are frequently expressed. From my own experience, quality of life is an umbrella term covering a wide variety of concepts. While an adult may declare that he would “rather die than end up in a wheelchair,” the parent of a child in the end stage of a progressive brain disease may say that there is still sometimes a smile on the face of her child and therefore the child's quality of life is good. In many instances, the quality of life of a severely incapacitated child fully depends on the irreplaceable care, love, and health of parents. When these “quasi inexhaustible resources” are lost due to the progress of time, when a patient's “child appeal” has disappeared or the parents have died, a patient's quality of life may strongly decrease.

*Comment. Ethical decisions about a patient*'*s quality of life should be considered as highly individual, which confers low priority to “moral” considerations (moral referring to supra-individual norms) from outside*.

### Families' Quest for New Therapies

NCL are incurable diseases, but at present, several novel targeted therapy approaches are emerging, and most parents of an affected child express a strong desire to participate at a study “at any price.” While eligibility of patients with rare diseases to participate in a clinical trial is a complex matter, families must understand some basic questions: the unpredictable outcome and risks of a newly proposed procedure in general, and the individual suitability of their child at the actual stage of the progressive disease.

*Comment. Advising a family in respect to a new treatment requires much personal judgement. A study in families with metachromatic leukodystroph (a progressive neurodegenerative disease with similarities to NCL) showed that preservation of speech or active communication, as well as a stop of disease progression, had the highest parental priorities expected for new treatments* *(**14**)*.

## Aspects Related to Novel Therapies

Recently, clinical trials that were performed under rigidly controlled circumstances have shown that enzyme replacement therapy (ERT) in patients with CLN2 disease can significantly slow down the progressive deterioration of neurological function (15). Not surprisingly, this exciting achievement is fraught with new challenging ethical questions.

### Experimental Therapies Outside Clinical Trials

Once a new treatment has been shown to be safe and efficacious and has been approved by the regulatory authorities, it remains experimental for some time for a variety of reasons. In the case of ERT for CLN2 disease, which involves repeated complex invasive procedures for drug delivery to the brain (15), one of the remaining questions is how the results will look like when the treatment is used under less strictly controlled conditions. Such observations have been made in France (16) and in Colombia (17) but do not suffice yet in this respect.

*Comment. Once an experimental drug is selling well, the producing company*'*s interest to critically follow late results of treatment may fade away. Whose obligation is it then to take over responsibility?*

### New Targeted Therapies—Are They Only Disease-Modifying?

Another aspect of novel treatments in severe rare diseases causes much greater concern. While enzyme replacement therapy for CLN2 disease effectively slows neurological progression, there are no long-term results yet (15). In the waiting time, we have to make efforts to shift the time point of diagnosis to younger ages, as an early start of treatment, ideally in pre-symptomatic children, will most probably have an impact on the results.

**Figure 1:**
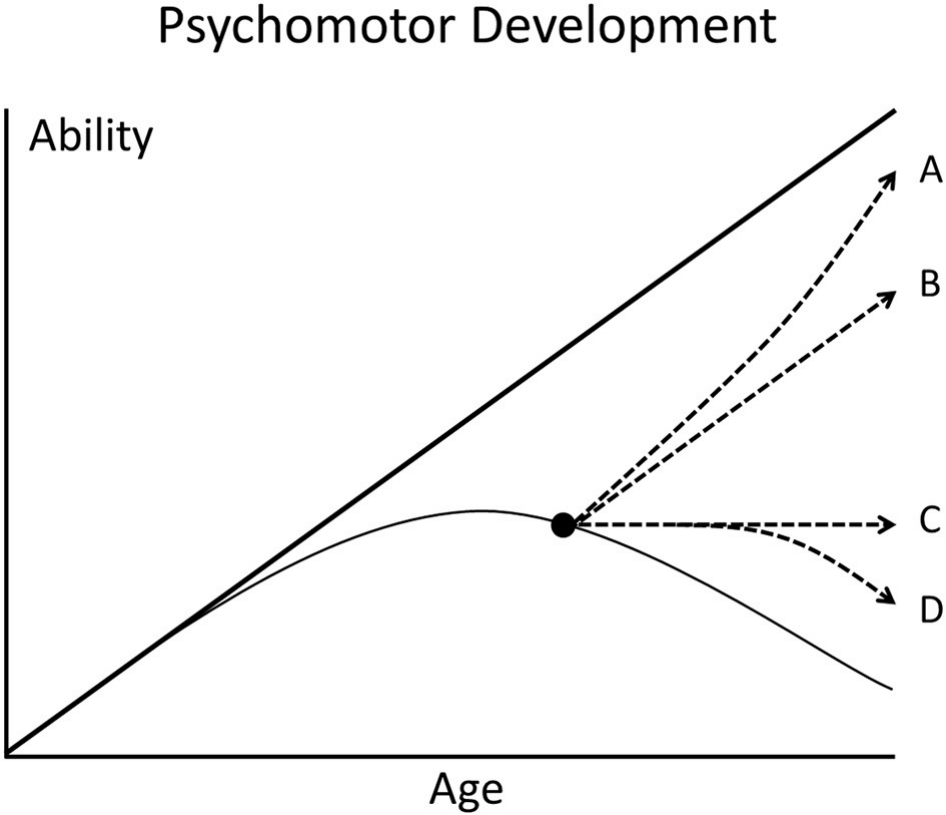
Scheme of the psychomotor development of children. The thick straight line represents the development of a healthy child who steadily gains abilities with increasing age. The thin curved line represents a child with degenerative brain disease. When effective therapy is started after appearance of symptoms (black dot), different deviations from the natural course of disease are possible (broken lines A–D).

In the meantime, clinicians and researchers have to live with an uncomfortable risk. Untreated late infantile CLN2 disease leads to loss of all human abilities and death at about 10 years of age. Enzyme replacement treatment delays the losses of mental and motor function (not that of vision) significantly but with unknown psychomotor functions later in life. It may be that treatment modifies a terrible disease with short life expectancy into a long life with possibly poor quality of life (Figure 1).

Analogous problems have occurred with other neurodegenerative disorders of childhood. Infantile Krabbe disease is a lysosomal degenerative brain disease that leads to death within the first 2 years of life. Hematological stem cell transplantation can curb the progressive brain destruction and leads, when performed shortly after birth, to prolonged survival and achievement of more psychomotor abilities than untreated patients can obtain. The overall results of treatment, however, have been disappointing (18). Long before this became recognizable and other important facts being also unknown, newborn screening for Krabbe disease was enthusiastically introduced in the state of New York in 2006. This project has been associated with many false hopes and binding of enormous resources (19).

Spinal muscular atrophy (SMA), a progressive neuromuscular disorder, is caused by a gene defect leading to the dying off of motoneurons. Infants with the SMA1 type mostly die within the first 2 years of life or require ventilation >16 h per day. Recently developed genetically targeted therapies drastically alter the rapid progression of the disease in the 1st years of life; the later course of disease remains uncertain (20) as longitudinal data are outstanding. Extraordinarily high costs (drug prices, personnel resources, etc.) in the context of limited available evidence are calling for a just balance of interests of patients, healthcare systems, pharmaceutical industry, and society (21).

*Comment. Clinical research has to take risks to achieve therapeutic progress but may contemporaneously create new problems by disease-modifying therapies. We must continue to analyze what we are doing and define responsibilities*.

### Newborn Screening

The principle of newborn screening for treatable rare diseases is detecting them in a preclinical stage and preventing the outbreak of symptoms by early intervention. Given the treatability of CLN2 patients by enzyme replacement therapy (15), the disease becomes an applicant for inclusion into routine newborn screening. Feasibility and reliability of testing for the disease in dried blood samples for use with screening have been demonstrated (22).

The “classic” view of the appropriateness of newborn screening for a disease is that it can be treated effectively. Due to the lack of long-term data on enzyme replacement therapy in CLN2 patients, this disease at present does not qualify for newborn screening. Recently, a “non-classic” view has argued that screening is appropriate even for diseases without available treatment. Proponents of this view list as values of such an expanded screening that it gives parents diagnostic and prognostic information about their child, allows them to make more informed reproductive decisions, improve symptomatic therapies, and will stimulate research on poorly understood diseases (23). Implementing an expanded screening of this kind appears at present impossible in Europe, given legal and other restrictions. Nevertheless, studies of newborn screening for several lysosomal storage disorders (24), among them for mucopolysaccharidoses (25, 26), have documented their feasibility and have investigated ethical, political, and other remaining obstacles for including these diseases into general screening programs.

*Comment. A more liberal view of newborn screening that would include diseases with questionable or even absent treatability bears some attractivity. Among the obstacles of implementation are the complexity of an imperative prenatal education* *(**27**)* *and respect for a “right not to know”* *(**28**)*.

### Public Health and General Research Issues

NCL belong to the thousands of diseases for which the fact of their rarity adds to suffering of patients and hampers the development of treatments. Rarity leads to insufficient knowledge of the course of disease and of disease mechanisms and to too few participants in clinical trials of proposed new therapies (29, 30). Rarity creates a host of questions for stakeholders in the field: affected patients with their families, patient organizations and supporting foundations, healthcare providers (medical services and pharmaceutical industry), insurance or other payments systems, public health policymakers, and research institutions.

Clinical research faces the impact of a set of rare disease characteristics that influence the methodology of completing robust studies (29). A study of the perspectives of different stakeholders on therapy-related research concluded that stakeholders have divergent views on rare disease research but share concerns about the risks vs. benefits of therapies when making their decisions (31).

Development of drugs or procedures for rare diseases and their pricing is causing much debate. An extreme situation was reached when an individualized drug therapy with an allele-specific oligonucleotide was developed for a single patient suffering from a type of NCL caused by a specific rare mutation of the *CLN7* gene (32). A systematic review of ethical problems linked to rare diseases and orphan drugs lists the following major issues: the funding of research, the significance of non-economic values like compassion and beneficence in decision-making, the identification of limits to labeling diseases as rare, barriers to supranational cooperation, and determining panels of decision-makers (33). Rare disease policies and reimbursement systems for orphan medicinal products and healthcare services differ greatly between countries (34, 35).

In a process known as venture philanthropy, private foundations obliged to specific diseases have formed partnerships with industry and federal agencies to share the financial risk of therapeutic development (36). For three lysosomal storage disorders, a charitable access program for patients in underserved communities worldwide has been instituted (37). This program could become a model for cooperation between industry, patient organizations, and governmental and non-governmental organizations in fighting rare diseases.

*Comment. The rarity alone of a disease causes a multitude of problems. As 3–6% of the world population are affected by thousands of rare diseases* *(**38**), the potential load required by these diseases for healthcare and research is astronomical*.

### Further Topics

In NCL, as in many genetic devastating diseases, professionals will be confronted with further topics requiring difficult ethical decisions: prenatal diagnosis, pre-implantation diagnosis, or results of carrier screening as determinants of reproductive choices (39). These are beyond the scope of this review.

## Discussion

Difficult questions related to NCL or similar diseases are manifold and have been presented here as forming two major groups. One group centers on the care of individual patients on their way to dementia, complete helplessness, and early death. Advanced care planning for the end of life and assumptions on the best interest of a patient constitutes one of the most challenging problems. A second group of problems mainly concerns experimental, targeted therapies. New therapies, even when astonishingly effective, carry the risk of being disease-modifying with a potentially undesirable outcome. The sections in the text on single problem areas are followed by short personal comments that try to mark particularly hot spots of discussion (printed in *italics*).

In conclusion, the ethical issues presented should make physicians and other professionals, including researchers and politicians, aware of having to take their own decisions in widely different situations caused by progressive brain diseases of young persons, as they typically occur in NCL.

## Author Contributions

The author confirms being the sole contributor of this work and has approved it for publication.

## Conflict of Interest

The author declares that the research was conducted in the absence of any commercial or financial relationships that could be construed as a potential conflict of interest.
